# Advances in Computational Genomics

**DOI:** 10.1155/2015/187803

**Published:** 2015-01-06

**Authors:** Leng Han, Yan Guo, Zhixi Su, Siyuan Zheng, Zhixiang Lu

**Affiliations:** ^1^Department of Bioinformatics and Computational Biology, University of Texas MD Anderson Cancer Center, Houston, TX 77030, USA; ^2^Department of Cancer Biology, Vanderbilt University, Nashville, TN 37232, USA; ^3^State Key Laboratory of Genetic Engineering and MOE Key Laboratory of Contemporary Anthropology, School of Life Sciences, Fudan University, Shanghai 200433, China; ^4^Department of Microbiology, Immunology & Molecular Genetics, University of California, Los Angeles, CA 90095, USA

High-throughput technologies, such as microarray and next generation sequencing (NGS), have emerged as a powerful tool less than a decade ago and have produced an avalanche of genome sequences. Computational genomics, which focus on computational analysis from genome sequences to other postgenomic data, including both DNA and RNA sequences, protein profiling, and epigenetic profiling, have become one of the most important avenues for biological discovery. Transforming genomic information into biomedical and biological knowledge requires creative and innovative new computational methods for all aspects of genomics. In this special issue, we selected fifteen high-quality papers in computational genomics field after in-depth peer review, and we briefly described these papers below.

Z. Su et al. performed an extensive analysis of the mouse knockout phenotype data and corroborated a strong effect of duplicate genes on mouse genetics robustness. The study suggested the potential correlation between the effect of genetic buffering and sequence conservation as well as protein-protein interactivity.

Y. Guo et al. developed a software package, O18Quant, which calculated the peptide/protein relative ratio and provided a friendly graphical user interface (GUI). The software greatly enhanced user's visualization and understanding in quantitative proteomics data analysis.

S. Zhao et al. developed an R package, “heatmap3,” which significantly improved the original “heatmap” function by adding several more powerful and convenient features, including highly customizable legends and side annotation, a wider range of color selections, and new labeling features.

W. Chen et al. developed a newly developed software package, Genepleio, to estimate the effective gene pleiotropy from phylogenetic analysis of protein sequences. This work would facilitate the understanding of how gene pleiotropy affected the pattern of genotype-phenotype map and the consequence of organismal evolution.

S. Lee at al. developed the BLAST-like alignment tool (BLAT) based comparative analysis for transposable elements (BLATCAT) program and compared specific regions of representative primate genome sequences.

H. Zhang et al. developed a new method for tumor-gene selection, the chi-square test-based integrated gene rank and direct classifier. The informative genes selected significantly improved the independent test precision of other classifiers.

L. Zhong et al. analyzed miRNA-mRNA paired variations (MMPVs) comprehensively and demonstrated that the existence of MMPVs is general and widespread but that there is a general unbalance in the distribution of MMPVs among the different pathological features.

N. Jin et al. systematically evaluated frequently used methods using two types of integration strategies, empirical and machine, and provided an important basis for future network-based biological research learning methods.

H. Zhao et al. developed an integrated strategy to identify differential coexpression patterns of genes and probed the functional mechanisms of the modules. This approach was able to robustly detect coexpression patterns in transcriptomes and to stratify patterns according to their relative differences.

A. V. Polonikov et al. comprehensively analyzed the associations between adult asthma and single nucleotide polymorphisms and found the epistatic interactions between ADE genes underlying asthma susceptibility and the genetic heterogeneity between allergic and nonallergic variants of the disease.

J. Shu et al. constructed a database to design vaccine that targeted the Chinese, and predicted 20 potential HIV epitopes. This work will facilitate the development of a CD4+ T cell vaccine especially catered for the Chinese.

Y. Li et al. built a link map between small molecules and pathways using gene expression profiles, pathways, and gene expression of cancer cell lines intervened by small molecules and provided a valuable reference for identifying drug candidates and targets in molecularly targeted therapy.

Y.-D. Gao et al. investigated the* E. coli* strains in the human gut microbiome by using deep sequencing data. The authors reconstructed genome-wide metabolic networks for the three most common* E. coli* strains and provided a systematic perspective on* E. coli* strains in the human gut microbiome.

T. Tan et al. compared small RNA signatures to address small RNA transition during mouse spermatogenesis and provided insight into the mechanisms involved in the regulation of spermatogonial stem cells activities.

H. Hu et al. performed RNA-seq to investigate the mice testicular transcriptome to elucidate the mechanism of male reproductive toxicity and found several transcriptional signatures closely related to the biological processes of regulation of hormone, gamete generation, and sexual reproduction.

By launching this issue, we wish to stimulate the continuing efforts in computational genomics for more efficient analysis of big genomics data.

